# Associations between apolipoprotein B and bone mineral density: a population-based study

**DOI:** 10.1186/s12891-023-06990-x

**Published:** 2023-11-02

**Authors:** Xuefei Zhao, Ning Tan, Ya Zhang, Mengde Xiao, Lihong Li, Zhongxing Ning, Mingjiang Liu, Haimin Jin

**Affiliations:** 1 Department of Spine Surgery, The Affiliated Second Hospital, Hengyang Medical School, University of South China, Hengyang, 421009 China; 2 Department of Urology, The Affiliated Second Hospital, Hengyang Medical School, University of South China, Hengyang, 421009 China; 3 Department of Gland Surgery, The Affiliated Nanhua Hospital, Hengyang Medical School, University of South China, Hengyang, 421002 China; 4 Department of Intensive Care Unit, The People’s Hospital of Guangxi Zhuang Autonomous Region & Research Center of Intensive Care Unit, Nanning, 530021 China; 5 Department of Hand & Microsurgery, The Affiliated Nanhua Hospital, Hengyang Medical School, University of South China, Hengyang, 421002 China; 6 Department of Neurology, Wenzhou Central Hospital, Dingli Clinical Institute of Wenzhou Medical University, Wenzhou, 325000 China

**Keywords:** Apolipoprotein B, Bone mineral density, NHANES, Osteoporosis, Cross-sectional study

## Abstract

**Background:**

Lipids are critical in bone metabolism, and several studies have highlighted their importance. This study aimed to investigate the relationship between apolipoprotein B (apo B) and bone mineral density (BMD) at different skeletal sites (lumbar spine, femoral neck, and total femur) and to compare the influence of apo B with other traditional lipid markers.

**Methods:**

The study included participants from the National Health and Nutrition Examination Survey (NHANES) between 2011 and 2016 who had complete data for apo B and BMD at the three skeletal sites. We used weighted multivariate regression analysis, subgroup analysis, and interaction tests to examine associations. Restricted cubic spline (RCS) was used to examine the non-linear relationship.

**Results:**

A total of 4,258 adults were included in the study. Multivariate linear regression analysis showed that the relationship between apo B and BMD varied by skeletal site: a negative association was found with lumbar spine BMD [β = -0.054, 95%CI: (-0.073, -0.035)]. In contrast, a positive association was found with femoral neck BMD [β = 0.031, 95%CI: (0.011, 0.051)] and no significant association between apo B and total femur BMD.

**Conclusions:**

Our findings suggest that apo B is associated with BMD in a site-specific manner.

**Supplementary Information:**

The online version contains supplementary material available at 10.1186/s12891-023-06990-x.

## Background

Osteoporosis, a chronic disease that results in a decrease in bone mass and bone mineral density (BMD), poses a major health concern globally. Characterized by fragile bones and an increased susceptibility to bone disorders, it is estimated that approximately 200 million individuals are afflicted with osteoporosis or osteopenia [1, 2]. While aging is a significant risk factor, lifestyle and nutritional influences also contribute to the development and progression of the disease [3, 4].

Lipids play a vital role in various physiological processes, including bone metabolism. Several studies have underscored the influence of lipids on bone health [5], indicating a potential association between specific lipid components, such as apolipoprotein B (apo B), and BMD [6]. Apo B, a primary component of atherogenic lipoproteins with two major isoforms: apo B48 and apo B100 [7], is implicated in numerous metabolic processes besides cholesterol transport. Notably, apo B is involved in several inflammatory pathways such as the nuclear factor kappa-light-chain-enhancer of activated B cells (NF-κB) pathway and the mitogen-activated protein kinases (MAPK) pathway [8, 9]. Its direct involvement in bone metabolism, however, remains largely unexplored, which our study aims to address.

Given apo B's role as a marker for cardiovascular risk [10] and its potential impact on bone health, it is vital to examine the relationship between apo B levels and BMD. While it has been suggested that lipids, including apo B, may play a role in bone health [11], to the best of our knowledge, no research has directly investigated this relationship. This leaves a knowledge gap in understanding the factors influencing bone health. To contribute to fill this gap, our study seeks to investigate the association between apo B levels and lumbar BMD within the framework of the National Health and Nutrition Examination Survey (NHANES).

## Methods

### Study population

**Fig. 1:**
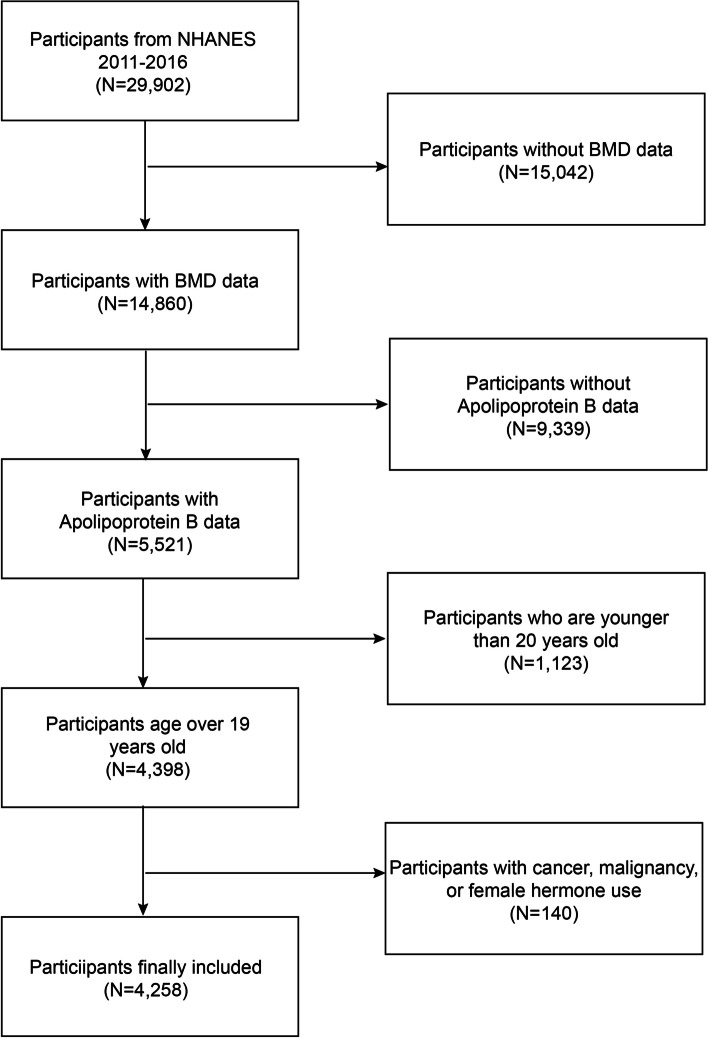
Flow chart of participants selection. NHANES, National Health and Nutrition Examination Survey; BMD, bone mineral density

The NHANES is a comprehensive survey that provides valuable information on the health and nutritional status of the US population. It uses a complex, multistage, and probabilistic sampling process to ensure that the sample is representative of the overall population [12]. For this study, we used data from the 2011–2016 continuous cycle of the NHANES dataset. To ensure the quality of the data, we excluded participants with missing lumbar BMD data (*n* = 15,042), missing apo B data (*n* = 9,339), those younger than 20 years old (*n* = 1,123), and those with cancer, malignancy, or female hormone use (*n* = 140) from the initial sample of 29,902 eligible individuals. The final sample included 4,258 participants. The flow chart of the sample selection process is presented in Fig. 1. All human subjects involved in this study were treated in accordance with the ethical principles outlined in the Declaration of Helsinki, and the study was approved by the Research Ethics Review Board of the National Center for Health Statistics (NCHS).

## Study variables

In this study, we investigated the relationship between apo B and lumbar BMD, which was the dependent variable of interest. A light scattering immunochemical method was used to measure the concentration of apo B in human serum samples. In this assay, apo B in the sample forms immune complexes with specific antibodies. The formation of these complexes scatters a beam of light passed through the sample. The intensity of the scattered light is directly related to the concentration of apo B present. This method is a well-accepted and reliable technique for apo B measurement. Apo B, as the primary protein constituent of LDL and accounting for approximately 95% of LDL's total protein content, is integral to cholesterol transportation from the liver to vessel cells. Hence, elevated levels of Apo B, which can be accurately measured through the described method, often indicate atherosclerotic vascular changes, thereby serving as a risk factor for atherosclerosis. The quantification of apo B in this study is evaluated by comparison with a standard of known concentration.

Dual-energy X-ray absorptiometry (DXA) was used to quantify BMD in the lumbar, whole femur, and femoral neck. Total femur and lumbar spine BMD measurements were taken using the Hologic QDR-4500A fan-beam densitometer in 2017–2018, and with the Hologic Discovery A densitometers (Hologic, Inc., Bedford, MA) in 2013–2014. Only lumbar BMD measurements were taken with a Hologic Discovery A densitometer (Hologic, Inc., Bedford, MA) in 2011–2012 and 2015–2016. The APEX was used to assess femur and lumbar images from 2011 to 2014 and 2015 to 2018. As previously verified, there were no significant differences in mean BMD values when analyzed using Hologic Discovery and APEX [13]. A rigorous quality control program was implemented for DXA measurements throughout the study, ensuring that the coefficient of variation for DXA measurements was less than 1%.

The covariates in our study were selected based on previous literature, which includes gender, age, race, education level, and family income-to-poverty ratio have been shown to be associated with BMD in various studies [14, 15]. Similarly, body mass index is well-known factors related to bone health and lipid metabolism [16, 17]. The laboratory variables we included, such as alanine aminotransferase, aspartate aminotransferase, alkaline phosphatase, total calcium, serum phosphorus, and globulin, are standard tests for assessing general health status and have been linked to BMD and/or lipid levels in previous research [18]. Finally, the questionnaire variables, including smoking status, alcohol drinking status, moderate physical activities status, prescription for cholesterol, diabetes status, and arthritis status, are lifestyle and health factors that are often considered in epidemiological studies of BMD and lipid metabolism [19, 20].

## Statistical analysis

We used R version 4.1.3 and Python version 3.10.4 to conduct the statistical analysis in this study. Multiple imputation was used to handle missing BMD and covariate data. Descriptive statistics for the baseline characteristics of the study population were presented using apo B subgroups for categorical variables and mean values with standard deviations for continuous variables. Weighted linear regression models were used to account for the complex survey design. We used multivariate linear regression analysis to estimate the beta values and 95% confidence intervals for the association between apo B and lumbar BMD. Three models were created: Model 1 included no covariates, Model 2 adjusted for gender, age, and BMI, and Model 3 adjusted for all covariates. Stratified analyses were conducted for gender, age, race, education level, BMI, smoking status, drinking status, diabetes status, exercise status, and arthritis status. Interaction tests were used to investigate differences in associations across populations, the *P*-values for interaction were examined by likelihood ratio tests. Restricted cubic spline (RCS) was applied to visualize the association between apo B and BMD. Correlation matrix heatmap was created to visually represent the relationships between Apo B, other traditional lipid markers, and BMD at different skeletal sites. Statistical significance was set at *P* < 0.05.

## Results

### Baseline characteristics

Table 1 displays the clinical features of the study sample, categorized by apo B quartiles. The study encompassed 4,258 participants who fulfilled the inclusion and exclusion criteria, with an average age of 37.56 ± 12.31 years. Among these participants, 52.02% were male, 47.98% were female, and 23.27% of the females were menopausal. The racial/ethnic composition included 35.04% non-Hispanic white, 21.54% non-Hispanic black, 15.10% Mexican American, and 28.32% from other racial/ethnic backgrounds (Table 1).

**Table 1:** Weighted characteristics of the study population based on apolipoprotein B quartiles

Apolipoprotein B (g/L)	Total	Q1 (≤ 0.70)	Q2 (0.71–0.87)	Q3 (0.88–1.04)	Q4 (≥ 1.05)	*P*-value
	*N* = 4,258	*N* = 1014	*N* = 1100	*N* = 1038	*N* = 1106	
Age (years)	37.555 ± 12.312	32.878 ± 12.063	36.894 ± 12.192	40.022 ± 11.795	43.481 ± 10.158	< 0.001
Sex, (%)						< 0.001
Male	52.020	47.561	49.789	51.799	61.100	
Female	47.980	52.439	50.211	48.201	38.900	
Postmenopausal female	11.165	8.542	10.419	12.498	11.716	
Premenopausal female	36.815	43.897	39.792	35.703	27.184	
Race/Ethnicity (%)						0.037
Non-Hispanic White	35.040	60.395	61.974	61.937	63.407	
Non-Hispanic Black	21.536	14.152	11.881	9.941	9.652	
Mexican American	15.101	8.944	10.126	11.198	11.692	
Other race/ethnicity	28.323	16.509	16.020	16.925	15.249	
Education level (%)						< 0.001
Less than high school	15.176	12.818	14.757	14.933	18.195	
High school	20.229	19.163	18.237	19.882	23.635	
More than high school	64.595	68.019	67.006	65.185	58.170	
Moderate activities (%)						0.048
Yes	43.776	49.854	47.320	45.134	44.159	
No	56.224	50.146	52.680	54.866	55.841	
Smoked at least 100 cigarettes in life, n (%)						< 0.001
Yes	39.027	33.536	41.124	40.876	46.996	
No	60.973	66.464	58.876	59.124	53.004	
Had at least 12 alcohol drinks in 1 year, n (%)						0.010
Yes	73.258	76.667	79.409	78.790	81.705	
No	16.742	23.333	20.591	21.210	18.295	
Diabetes, n (%)						0.008
Yes	6.881	8.531	5.194	5.670	7.776	
No	93.119	91.469	93.010	92.708	89.805	
Take prescription for cholesterol, (%)						0.019
Yes	8.196	6.410	7.818	8.285	10.127	
No	91.804	93.590	92.182	91.715	89.873	
Arthritis, n (%)						< 0.001
Yes	13.434	9.758	13.920	15.647	18.281	
No	86.564	90.242	86.080	84.353	81.719	
Body mass index (kg/m^2^)	28.761 ± 7.102	26.508 ± 6.762	28.541 ± 6.989	29.683 ± 6.805	30.695 ± 6.670	< 0.001
Income to poverty ratio	2.409 ± 1.662	2.6766 ± 1.6927	2.8033 ± 1.6928	2.9478 ± 1.6961	3.1525 ± 1.7327	< 0.001
ALT (U/L)	26.133 ± 19.656	21.376 ± 14.146	25.851 ± 21.278	27.169 ± 18.666	30.586 ± 18.620	< 0.001
AST (U/L)	25.700 ± 24.285	24.043 ± 24.677	25.962 ± 25.753	25.357 ± 14.509	27.010 ± 14.964	0.010
ALP (U/L)	66.030 ± 22.041	61.745 ± 21.678	63.438 ± 20.193	65.566 ± 19.744	69.127 ± 27.203	< 0.001
Total calcium (mg/dL)	9.353 ± 0.329	9.325 ± 0.312	9.338 ± 0.326	9.341 ± 0.336	9.390 ± 0.332	< 0.001
Serum phosphorus (mg/dL)	3.729 ± 0.558	3.767 ± 0.561	3.722 ± 0.563	3.683 ± 0.539	3.638 ± 0.519	< 0.001
Globulin (g/dL)	2.868 ± 0.432	2.717 ± 0.410	2.785 ± 0.423	2.792 ± 0.404	2.831 ± 0.409	< 0.001
Lumbar BMD (g/cm^2^)	1.031 ± 0.150	1.057 ± 0.151	1.038 ± 0.152	1.021 ± 0.147	1.252 ± 0.160	< 0.001
Total femur BMD (g/cm^2^)	0.977 ± 0.141	0.981 ± 0.153	0.981 ± 0.153	0.962 ± 0.143	0.994 ± 0.124	0.027
Femoral neck BMD (g/cm^2^)	0.814 ± 0.138	0.813 ± 0.152	0.825 ± 0.152	0.804 ± 0.139	0.816 ± 0.120	0.502

Mean ± SD for continuous variables: the *P* value was calculated by the weighted linear regression model

(%) for categorical variables: the *P* value was calculated by the weighted chi-square test

*Abbreviation*: *BMD* bone mineral density, *ALT* alanine aminotransferase, *AST* aspartate aminotransferase, *ALP* alkaline phosphatase, *HDL-C* high density lipoprotein cholesterol, *LDL-C* low-density lipoprotein cholesterol

Participants in the highest apo B quartile demonstrated a higher likelihood of being male, older, non-Hispanic white, or Mexican American. Additionally, they exhibited increased prevalence of arthritis, elevated rates of smoking, alcohol consumption, use of cholesterol prescription and higher levels of BMI, family income-to-poverty ratio, alanine aminotransferase, aspartate aminotransferase, alkaline phosphatase, total calcium, globulin, and total femur BMD. However, they presented lower levels of prevalence of diabetes, educational attainment, serum phosphorus, and lumbar BMD (*P* < 0.05).

**Fig. 2:**
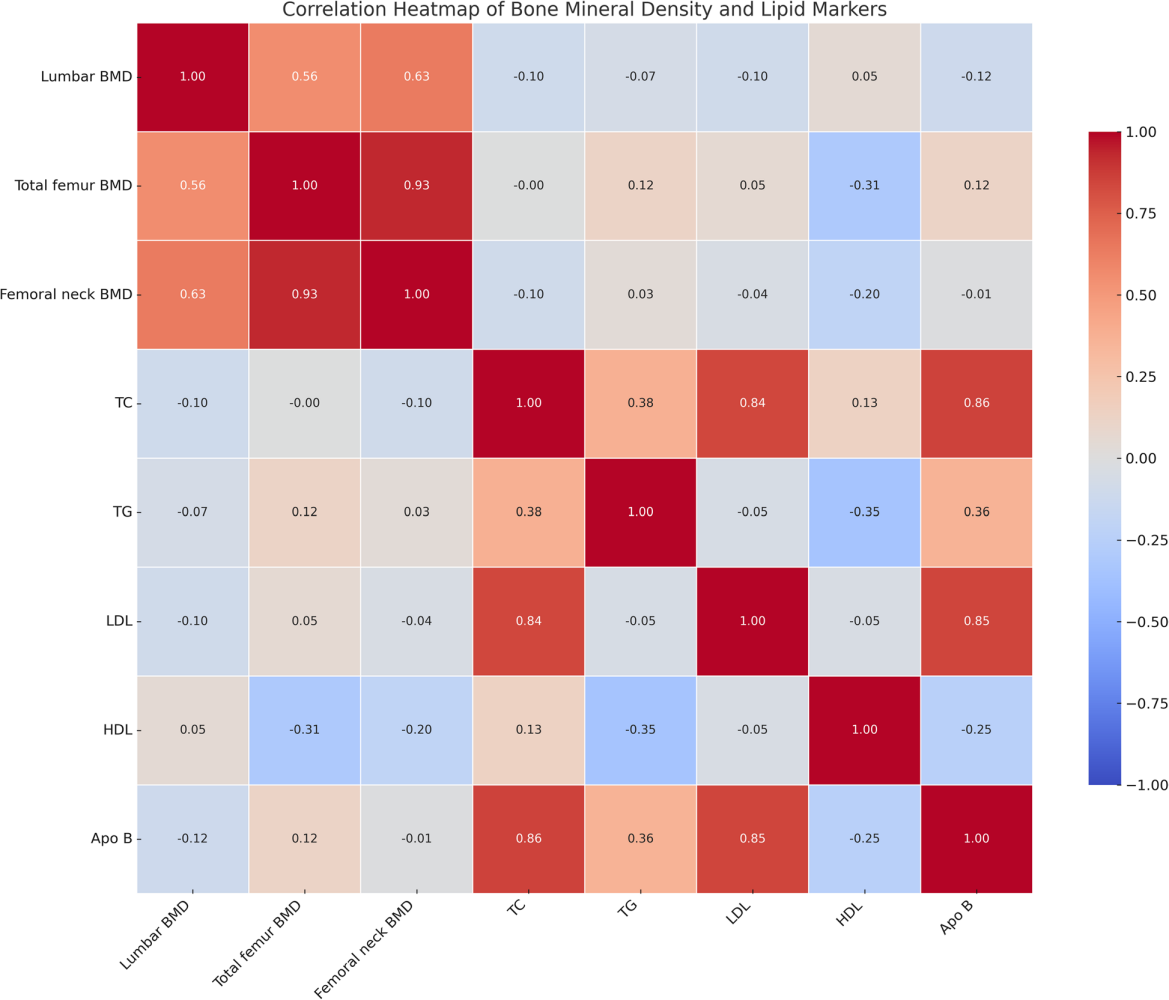
Correlation heatmap of bone mineral density and lipid markers. This heatmap illustrates the pairwise correlation coefficients between various bone mineral density measurements and lipid markers. The color intensity and the numeric values within each cell represent the strength and direction of the correlation. Darker shades of blue indicate a positive correlation, while darker shades of red indicate a negative correlation. The closer the value is to 1 or -1, the stronger the positive or negative correlation, respectively

In addition to the primary analyses, a correlation matrix heatmap was generated to visually represent the relationships between lipid biomarkers and BMD at different skeletal sites. Figure 2 shows the correlation coefficient between apo B, other lipid indicators, and BMD. There is significant collinearity between apo B, TC, and LDL-C.

## Association between apo B and BMD

**Fig. 3:**
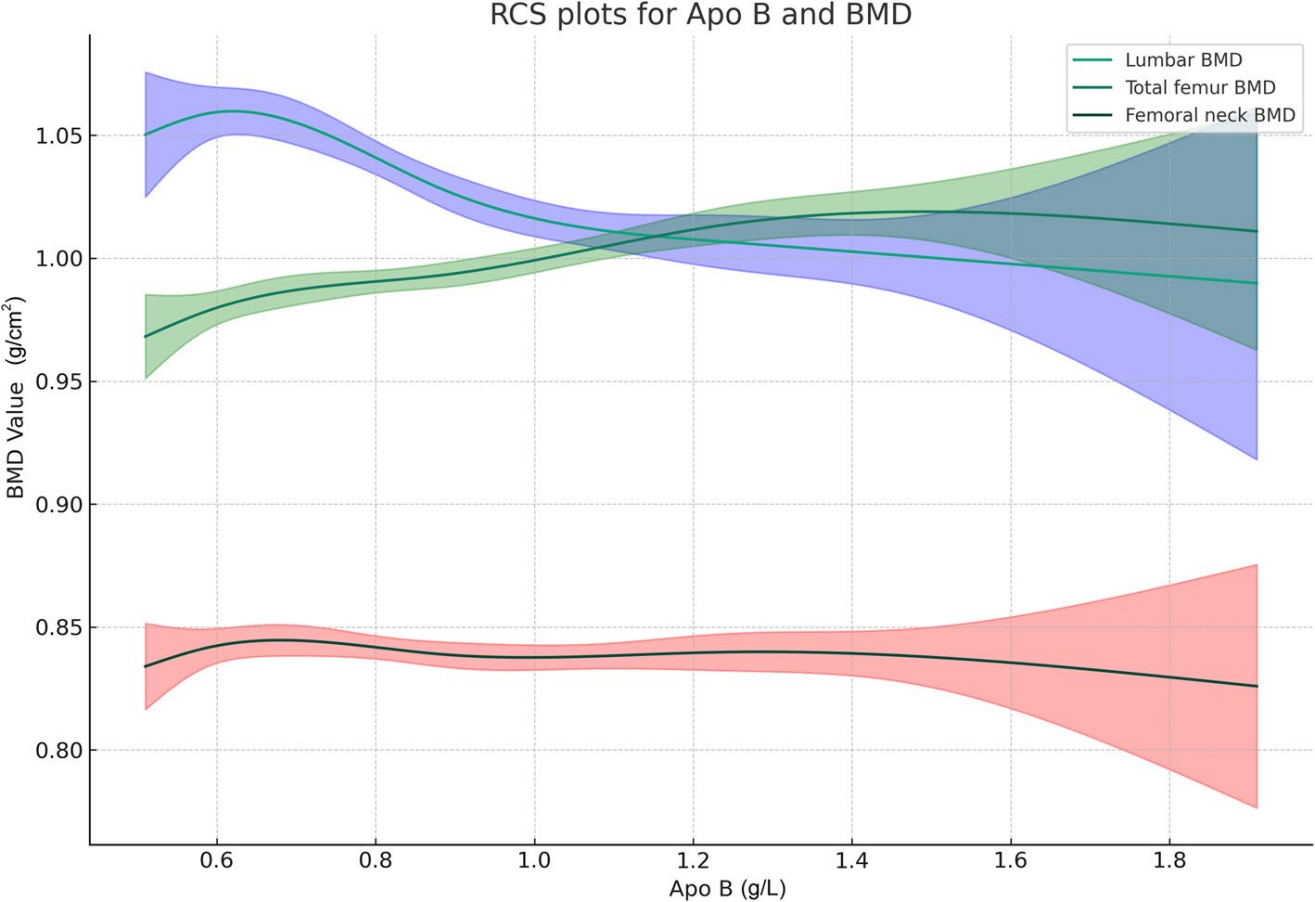
The nonlinear associations between apo B and bone mineral density. The solid line represents the smooth curve fit between variables. Translucent bands represent the 95% of confidence interval from the fit

Table 2 presents the associations between Apo B and BMD at three sites: lumbar, femoral neck, and total femur. In the unadjusted and minimally adjusted models, Apo B was negatively correlated with BMD at all three sites. For lumbar BMD, the fully adjusted model, after accounting for various covariates, revealed a negative correlation, with each unit increase in apo B associated with a decrease of 0.054 g/cm^2^ in lumbar BMD [β = -0.054, 95%CI: (-0.073, -0.035)]. Upon stratifying apo B into quartiles, participants in the highest quartile had a 0.037 g/cm^2^ lower lumbar BMD compared to those in the lowest quartile [β = -0.037, 95%CI: (-0.051, -0.023)]. Participants in the middle two quartiles also experienced a greater loss of lumbar BMD relative to those in the lowest quartile. For femoral neck BMD, the fully adjusted model revealed a positive correlation, contrary to the unadjusted and minimally adjusted models. With each unit increase in apo B, femoral neck BMD increased by 0.031 g/cm^2^ [β = 0.031, 95%CI: (0.011, 0.051)]. When apo B was divided into quartiles, participants in the highest quartile demonstrated a 0.023 g/cm^2^ higher femoral neck BMD compared to those in the lowest quartile [β = 0.023, 95%CI: (0.011, 0.037)]. For total femur BMD, the association with apo B did not show statistical significance in all models. Additional file 1: Table S1 describes the results of the same analysis performed on the data set before imputation to avoid bias, and there was no significant difference in trend compared with the results after imputation. Figure 3 depicts the nonlinear association between apo B and BMD at different sites.

**Table 2:** The association between apolipoprotein B and bone mineral density

Exposure	Model 1 [β (95% CI)]	Model 2 [β (95% CI)]	Model 3 [β (95% CI)]
Lumbar BMD (continuous)	-0.067 (-0.085, -0.050)	-0.039 (-0.058, -0.021)	-0.054 (-0.072, -0.036)
Lumbar BMD (quartile)			
Quartile 1	Reference	Reference	Reference
Quartile 2	-0.019 (-0.032, -0.006)	-0.018 (-0.031, -0.001)	-0.013 (-0.026, -0.000)
Quartile 3	-0.036 (-0.049, -0.023)	-0.018 (-0.030, -0.002)	-0.026 (-0.039, -0.012)
Quartile 4	-0.049 (-0.062, -0.036)	-0.033 (-0.044, -0.018)	-0.037 (-0.050, -0.024)
P for trend	< 0.001	< 0.001	< 0.001
Femoral neck BMD (continuous)	-0.010 (-0.050, 0.031)	-0.083 (-0.161, -0.016)	0.031 (0.011, 0.051)
Femoral neck BMD (quartile)			
Quartile 1	Reference	Reference	Reference
Quartile 2	0.011 (-0.022, 0.045)	-0.040 (-0.075, -0.023)	0.016 (0.006, 0.031)
Quartile 3	-0.009 (-0.042, 0.024)	-0.045 (-0.080, -0.014)	0.021 (0.007, 0.035)
Quartile 4	0.034 (0.002, 0.067)	-0.095 (-0.167, -0.011)	0.023 (0.011, 0.037)
P for trend	< 0.001	< 0.001	0.003
Total femur BMD (continuous)	0.036 (-0.005, 0.078)	-0.001 (-0.038, 0.038)	-0.025 (-0.063, 0.013)
Total femur BMD (quartile)			
Quartile 1	Reference	Reference	Reference
Quartile 2	0.022 (-0.013, 0.056)	0.032 (0.000, 0.064)	0.015 (-0.014, 0.044)
Quartile 3	0.003 (-0.031, 0.036)	0.019 (-0.013, 0.050)	0.000 (-0.028, 0.028)
Quartile 4	0.034 (0.002, 0.067)	0.039 (0.008, 0.069)	0.016 (-0.012, 0.043)
P for trend	0.055	0.044	0.415

Model 1: no covariates were adjusted. Model 2: age, gender, and BMI were adjusted. Model 3: age, gender, race, educational level, BMI,family income-to-poverty ratio, moderate activities, smoking status, alcohol use status, take prescription for cholesterol, prevalence of diabetes, prevalence of arthritis, ALT, AST, ALP, total calcium, globulin, female postmenopausal status, and serum phosphorus were adjusted

## Subgroup analysis

Subgroup analyses were performed to investigate the relationship between apo B and BMD across various subgroups at three sites: lumbar, femoral neck, and total femur (Table 3). For lumbar BMD, a negative association between apo B and lumbar BMD was observed in most of the subgroups stratified by sex, race/ethnicity, body mass index, and age. For femoral neck BMD, a negative association between apo B and femoral neck BMD was observed only in the body mass index ≥ 30 kg/m^2^ group, whereas no significant association was observed in the other groups. For total femoral BMD, the association was not significant in any of the subgroups. Interaction tests showed no significant differences between these strata in the association of Apo B with BMD at the three sites (*P* for interaction tests were > 0.05).

**Table 3:** Subgroup analysis of the association between apolipoprotein B and bone mineral density

**Subgroup**	**Lumbar BMD** **[β (95%CI)]**	**P for interaction**	Total femur BMD **[β (95%CI)]**	**P for interaction**	**Femoral neck BMD** **[β (95%CI)]**	**P for interaction**
Sex		0.245		0.268		0.266
Male	-0.063 (-0.088, -0.037)		-0.008 (-0.056, 0.039)		-0.039 (-0.087, 0.010)	
Female	-0.040 (-0.068, -0.012)		0.030 (-0.020, 0.081)		0.001 (-0.050, 0.052)	
Race/ethnicity		0.475		0.441		0.565
Non-Hispanic White	-0.039 (-0.070, -0.008)		-0.019 (-0.073, 0.036)		-0.043 (-0.098, 0.012)	
Non-Hispanic Black	-0.081 (-0.120, -0.042)		-0.020 (-0.097, 0.057)		-0.034 (-0.113, 0.045)	
Mexican American	-0.060 (-0.112, -0.008)		0.040 (-0.052, 0.132)		-0.007 (-0.100, 0.086)	
Other race	-0.028 (-0.090, 0.035)		0.039 (-0.031, 0.110)		0.019 (-0.053, 0.090)	
BMI, kg/m^2^		0.335		0.056		0.100
< 25	-0.035 (-0.071, 0.002)		0.082 (-0.000, 0.164)		0.043 (-0.040, 0.127)	
25–29.9	-0.069 (-0.102, -0.036)		-0.035 (-0.089, 0.019)		-0.046 (-0.101, 0.009)	
≥ 30	-0.042 (-0.072, -0.011)		-0.038 (-0.098, 0.022)		-0.068 (-0.129, -0.007)	
Age, years		0.159		0.290		0.221
< 50	-0.060 (-0.081, -0.039)		0.024 (-0.023, 0.070)		-0.004 (-0.051, 0.043)	
≥ 50	-0.030 (-0.067, 0.006)		-0.014 (-0.066, 0.039)		-0.048 (-0.101, 0.006)	
Diabetes		0.128		0.610		0.476
Yes	0.006 (-0.058, 0.070)		-0.010 (-0.097, 0.076)		-0.050 (-0.138, 0.038)	
No	-0.056 (-0.077, -0.036)		-0.010 (-0.097, 0.076)		-0.015 (-0.054, 0.023)	

Age, gender, race, educational level, BMI,family income-to-poverty ratio, moderate activities, smoking status, alcohol use status, take prescription for cholesterol, prevalence of diabetes, prevalence of arthritis, ALT, AST, ALP, total calcium, globulin and serum phosphorus were adjusted

## Discussion

Our study, analyzing a nationally representative sample of US adults, demonstrated complex associations between apo B levels and BMD at three sites: lumbar, femoral neck, and total femur. While a negative correlation was identified between apo B and lumbar BMD, a positive correlation was observed for femoral neck BMD. This indicates that the influence of apo B may vary across different skeletal sites. Subgroup analyses further revealed that these associations could potentially be influenced by factors such as sex, race, age, BMI, and diabetes status.

To the best of our knowledge, this is the first study to investigate the association between apo B and BMD. Apo B is a lipoprotein involved in lipid transportation and serves as a precursor to atherosclerosis. It is commonly utilized as a predictor of cardiovascular risk [9]. For instance, a recent case–control study proposed that apo B may function as a potential biomarker for atrial fibrillation, potentially playing a role in the initiation and maintenance of the condition in conjunction with several metabolic factors [21]. In addition, Marston et al. reported that the amount of apo B lipoprotein, compared to other lipid indicators, was the best predictor of myocardial infarction risk [10].

Currently, numerous epidemiological studies have demonstrated the correlation between lipid biomarkers and BMD [5, 22]. A multicenter cross-sectional study conducted in China revealed that high LDL-C levels are an independent risk factor for bone loss in both men and women. Moreover, increasing age and menopause exacerbate the negative effects on bone mass in women [23]. Yang et al. utilized Mendelian randomization analysis to demonstrate a potential causal relationship between BMD and lipid profiles, including LDL-C, total cholesterol, triglycerides, and HDL-C [24]. Furthermore, LDL-C, a lipid biomarker significantly associated with apo B, has been shown to have a significant association with BMD in several studies [25, 26]. While past studies have concluded a negative association between LDL-C and BMD, some researchers have suggested a positive association or an invalid connection between LDL-C and BMD [27, 28]. A recent meta-analysis of ten studies found that individuals with osteoporosis had higher LDL-C levels than healthy controls [29]. This association may help elucidate the link between coronary vascular disease and osteoporosis, where high LDL-C levels are a critical risk factor [30]. Our findings indicate that the inverse relationship between apo B and BMD is consistent with evidence from numerous epidemiological studies on the association between LDL-C and BMD, as well as the relationship between coronary vascular disease and osteoporosis [31–33].

The underlying mechanism explaining the inverse association between apo B and BMD remains unclear. Several hypotheses have been proposed to explain this phenomenon. One potential explanation is that oxidized lipids, including oxidized apolipoproteins, may exert direct harmful effects on bone cells. These effects could lead to the inhibition of osteoblast differentiation and bone formation, the promotion of adipogenesis in mesenchymal stem cells at the expense of their osteogenic differentiation, and the induction of osteoclast differentiation and bone resorption [34, 35]. Another possibility is that high apo B levels may trigger an inflammatory response, and emerging evidence suggests that inflammation can negatively impact bone mass by altering osteoclast activation or function [36, 37]. However, direct evidence to support these hypotheses is lacking, and further research is needed to confirm these mechanisms.

While our study provides important insights, it also has several limitations that should be acknowledged. First, because this is a cross-sectional study, causality cannot be established. Additionally, despite adjusting for several relevant confounders, the possibility of residual confounding cannot be completely ruled out. Another important limitation is potential selection bias due to the exclusion of a substantial number of participants. This could have affected our results, and therefore, our findings should be interpreted with caution. Future research with more comprehensive inclusion criteria or strategies to minimize selection bias would be beneficial to verify and extend our findings. Despite these limitations, our study has several strengths. One key strength is the use of a nationally representative sample of US adults, making our findings generalizable to a diverse population. Additionally, the large sample size allowed for subgroup analyses, adding to the robustness of our results.

## Conclusion

Our study revealed a complex relationship between apo B levels and BMD at various sites. These findings highlight the unique role of apo B in bone metabolism and call for further investigations.

### Supplementary Information

**Additional file 1:** **Table S1.** The association between apolipoprotein B and bone mineral density before imputation.

## Data Availability

The datasets generated and analysed during the current study are available in the NHANES repository, [www.cdc.gov/nchs/nhanes/].

## Abbreviations

apo B: Apolipoprotein B
BMD: Bone mineral density
NHANES: National Health and Nutrition Examination Survey
RCS: Restricted cubic spline
ALT: Alanine aminotransferase
AST: Aspartate aminotransferase
ALP: Alkaline phosphatase
HDL-C: High-density lipoprotein cholesterol
LDL-C: Low-density lipoprotein cholesterol
NF-κB: Nuclear factor kappa-light-chain-enhancer of activated B cells
MAPK: Mitogen-activated protein kinases
