# Supplementary material for: Associations between apolipoprotein B and bone mineral density: a population-based study
Source: BMC Musculoskelet Disord. 2023 Nov 2;24:861. doi: 10.1186/s12891-023-06990-x (PMC10621203; doi:10.1186/s12891-023-06990-x)
Supplement: Supplementary file 1 — Additional file 1: Table S1. The association between apolipoprotein B and bone mineral density before imputation. [file 12891_2023_6990_MOESM1_ESM.docx]

**Table S1:** The association between Apolipoprotein B and bone mineral density before imputation.

| **Exposure** | **Model 1 [β (95% CI)]** | **Model 2 [β (95% CI)]** | **Model 3 [β (95% CI)]** |
| --- | --- | --- | --- |
| Lumbar BMD (continuous) | -0.067 (-0.085, -0.050) | -0.039 (-0.058, -0.021) | -0.054 (-0.072, -0.036) |
| Lumbar BMD (quartile) |  |  |  |
| Quartile 1 | Reference | Reference | Reference |
| Quartile 2 | -0.019 (-0.032, -0.006) | -0.018 (-0.031, -0.001) | -0.013 (-0.026, -0.000) |
| Quartile 3 | -0.036 (-0.049, -0.023) | -0.018 (-0.030, -0.002) | -0.026 (-0.039, -0.012) |
| Quartile 4 | -0.049 (-0.062, -0.036) | -0.033 (-0.044, -0.018) | -0.037 (-0.050, -0.024) |
| P for trend | <0.001 | <0.001 | <0.001 |
| Femoral neck BMD (continuous) | -0.010 (-0.052, 0.034) | -0.082 (-0.162, -0.013) | 0.032 (0.012, 0.053) |
| Femoral neck BMD (quartile) |  |  |  |
| Quartile 1 | Reference | Reference | Reference |
| Quartile 2 | 0.013 (-0.023, 0.041) | -0.040 (-0.075, -0.023) | 0.016 (0.006, 0.031) |
| Quartile 3 | -0.006 (-0.041, 0.026) | -0.046 (-0.081, -0.014) | 0.022 (0.007, 0.035) |
| Quartile 4 | 0.035 (0.002, 0.067) | -0.088 (-0.157, -0.012) | 0.025 (0.011, 0.038) |
| P for trend | 0.001 | <0.001 | 0.003 |
| Total femur BMD (continuous) | 0.035 (-0.005, 0.078) | -0.001 (-0.038, 0.038) | -0.025 (-0.062, 0.016) |
| Total femur BMD (quartile) |  |  |  |
| Quartile 1 | Reference | Reference | Reference |
| Quartile 2 | 0.022 (-0.013, 0.056) | 0.032 (0.000, 0.064) | 0.015 (-0.014, 0.043) |
| Quartile 3 | 0.003 (-0.031, 0.036) | 0.019 (-0.013, 0.050) | 0.001 (-0.025, 0.026) |
| Quartile 4 | 0.035 (0.002, 0.069) | 0.039 (0.008, 0.069) | 0.016 (-0.012, 0.043) |
| P for trend | 0.042 | 0.050 | 0.466 |

Model 1: no covariates were adjusted. Model 2: age, gender, and BMI were adjusted. Model 3: age, gender, race, educational level, BMI,family income-to-poverty ratio, moderate activities, smoking status, alcohol use status, take prescription for cholesterol, prevalence of diabetes, prevalence of arthritis, ALT, AST, ALP, total calcium, globulin, female postmenopausal status, and serum phosphorus were adjusted.
